# Progress in Psychiatry

**Published:** 1904-04-23

**Authors:** 


### Progress in Psychiatry.

(*Continued from Page* 43.)

**Psychical and Epileptoid Disturbances in Alcoholic Subjects.**—By application of the Roentgen method of photography, and with the use of the phonendoscope, Dr. A. Smith [6] has been able to demonstrate an increase in the size of the heart in every case of chronic alcoholism, sane and insane, examined by him. These patients are liable to mental disturbances of an insane and dangerous character as the consequence of epileptiform attacks, to which they are prone. Thus in one class of case the patient without any warning is seized with an attack of psychical epilepsy—a subacute delirium or confusion of mind attended with stupor ("twilight state of consciousness") which lasts several days. On recovery from this there is no recollection of events which occurred during this delirium stupor period, a phenomenon parallel to the post-epileptic automatism (with amnesia on subsequent recovery) which occurs in certain classes of epileptics. Other patients suffer from morning attacks of cardiac distress attended with slight epileptiform attacks; they are then driven to seek relief in alcohol, which is but temporary, and which often gives rise to a state of transitory delirium in which murder or suicide may be committed. A third class of cases consists of patients with a bad mental heredity, who, as the result of chronic or subacute alcoholic intoxication, have developed cardiac dilatation. After some weeks of treatment, with rest in bed, regulation of diet, and cardiac tonics, acute attacks of cardiac dilatation and distress supervene in the course of an apparent convalescence. These patients during the course of their illness become depressed in spirits and suspicious in disposition, the slightest thing upsets them, and a single dose of alcohol is liable to bring on an epileptiform fit. In alcoholic patients with dilated heart and a degree of mental disturbance not amounting to certifiable insanity, the best treatment, says Dr. Smith, is prolonged baths during the attacks of cardiac distress and slight mental delirium, outdoor exercise, Swedish movements pursued in the open air at intervals and so as not to produce fatigue, and the internal administration of trional and cardiac and nervine tonics.

**Hallucinatory Tendencies in Acute Alcoholism.**—Hallucinations of a dangerous and insane character readily develop in alcoholic subjects. Cololian and Rodiet [7] have published a lengthy study of such cases from Magnan's Clinic at St. Anne's Asylum, Paris. This tendency to illusions and hallucinations of the senses had been long known to exist in chronic alcoholic patients. Lipmann noted that visual hyperæsthesia existed, and that visual hallucinations could be readily induced in such subjects by closing the eyes and gently pressing on the sides of the eyeball. Krafft-Ebing has shown that merely closing the eyes will suffice to evoke visual hallucinations in some patients. Magnan has noted that taps on the skin can give rise to sensations (illusions) as of being bitten or stung by an insect, and that blowing into the external auditory meatus will give rise to auditory illusions and hallucinations.[8] Mierzejewski [9] observed that in alcoholic subjects there exists at the onset of delirium tremens such an excitability or hyperæsthesia of the visual centre that impressions of colour leave a long-persistent trace, and that hallucinations occurring at such moments take the same colour. He also observed that in alcoholic subjects tapping of the skin with a mallet provoked auditory hallucinations of "voices" uttering insults. Cololian [10] has in a recent thesis recorded instances of delusions of persecution arising from such simple hallucinations of touch and hearing. Similarly other senses are prone to act abnormally. Thus stimulating the tongue with a rolled piece of paper after closing the patient's eyes will provoke intense illusions of taste with grimaces of disgust. "One patient says he tasted sulphate of soda or quinine and made a horrid grimace, another said it was absinthe, a third said it was sugar, while a fourth experienced merely an agreeable taste which he could not define." The diagnosis of such cortico-sensorial hyperæsthia is of great practical importance, for it occurs before the actual onset of the symptoms of agitation, tremor, and acute cerebral excitement which characterise delirium tremens, and it is possible by prompt treatment to prevent or to mitigate the threatened attack. Of several cases quoted by Cololian and Rodiet the following may be cited as instructive: A man aged 32 years was arrested by the police and taken to the infirmary of the police depôt on June 13th, 1899. His maternal grandfather was an alcoholic inebriate, and his mother was a drinker of absinthe who indulged in dissipation and extravagance. He was the only child born to this woman. He suffered from convulsions when a child, and at about the age of 18 years began to take to drinking in excess. For 14 years afterwards he drank habitually and rather immoderately both absinthe and other spirituous liquors. He gradually developed symptoms of nervousness, insomnia with nocturnal hallucinations, tremors of the hands and cramps of the calves. In 1898 he was one night seized with an attack of delirium tremens. Before and during the first few days of his marriage (June 1898) he accused his wife of indifference, coldness, and deceit towards him, and said her mother connived at it. For three months after this he was sad and depressed, and spoke of shooting himself. He continued to drink as before, and on being examined after his arrest he was found to be in a state of tremor all over the body, with flushed face, brilliant eyes, and a face covered with sweat. The pulse was rapid, and there was cutaneous hyperæsthesia of the neck, chest, soles of the feet, and flexor surfaces of the forearm. "In all these hyperæsthetic areas the least rubbing produced agitation and contortions of the body with movements of defence, and the patient experienced actual pain." Tapping produced similar effects, and by Lipmann's method it was easy to provoke definite visual hallucinations of quivering flashes of light, sparks, and flames. Buzzing and roaring sounds were induced by simple suggestion or by blowing on the ears. Touching the tongue with paper induced tastes of "powder and of absinthe" so strong and nauseous that he attempted to vomit. Smell was normal. The cortical sensory hyperæsthesia and hallucinations subsided, but an increasing state of melancholia and gradual dementia supervened, rendering his detention in an asylum necessary.

[6] Münch Medicin. Wochenschr., No. 43, 1898, p. 1372. [7] Arch. de Neurol., May, 1900. [8] Leçons cliniques des Maladies mentales, 1897, p. 207. [9] Arch. Slaves de Biologie. March, 1886. [10] Les alcooliques persécutes, Thèse de Paris, 1897.

